# A simple protocol for cultivating the bacterivorous soil nematode *Caenorhabditis elegans* in its natural ecology in the laboratory

**DOI:** 10.3389/fmicb.2024.1347797

**Published:** 2024-02-27

**Authors:** Rocel Amor Indong, Jong Min Park, Jin-Kyung Hong, Eun Sun Lyou, Taeman Han, Jong Kwang Hong, Tae Kwon Lee, Jin I. Lee

**Affiliations:** ^1^Division of Biological Science and Technology, Yonsei University Mirae Campus, Wonju, Republic of Korea; ^2^Department of Environmental and Energy Engineering, Yonsei University Mirae Campus, Wonju, Republic of Korea; ^3^Korea National Park Research Insitute, Korea National Park Service, Wonju, Republic of Korea

**Keywords:** *C. elegans*, nematode ecology, soil ecology, soil microbe, ecological succession, bacterial predator, nematode cultivation

## Abstract

The complex interplay between an animal and its surrounding environment requires constant attentive observation in natural settings. Moreover, how ecological interactions are affected by an animal’s genes is difficult to ascertain outside the laboratory. Genetic studies with the bacterivorous nematode *Caenorhabditis elegans* have elucidated numerous relationships between genes and functions, such as physiology, behaviors, and lifespan. However, these studies use standard laboratory culture that does not reflect *C. elegans* true ecology. *C. elegans* is found growing in nature and reproduced in large numbers in soils enriched with rotting fruit or vegetation, a source of abundant and diverse microbes that nourish the thriving populations of nematodes. We developed a simple mesocosm we call soil-fruit-natural-habitat that simulates the natural ecology of *C. elegans* in the laboratory. Apples were placed on autoclaved potted soils, and after a soil microbial solution was added, the mesocosm was subjected to day-night, temperature, and humidity cycling inside a growth chamber. After a period of apple-rotting, *C elegans* were added, and the growing worm population was observed. We determined optimal conditions for the growth of *C. elegans* and then performed an ecological succession experiment observing worm populations every few days. Our data showed that the mesocosm allows abundant growth and reproduction of *C. elegans* that resembles populations of the nematode found in rotting fruit in nature. Overall, our study presents a simple protocol that allows the cultivation of *C. elegans* in a natural habitat in the laboratory for a broad group of scientists to study various aspects of animal and microbial ecology.

## Introduction

The diversity and density of soil microbial populations in nature and agricultural settings depend on a balance between abiotic and biotic factors. In particular, bacterivorous organisms, such as nematodes, predate on bacterial prey which can shape the microbial ecology in soil. In the same way, nematode survival and population growth depend on the abundance of their bacterial prey. How microbial and nematode populations affect each other in the delicate balance of soil ecology has not been fully characterized.

The free-living nematode *Caenorhabditis elegans* has been one of the most widely used genetic model organisms leading to seminal genetic discoveries. The *C. elegans* genome was the first multicellular organism sequenced in 1998, and thousands of studies using *C. elegans* mutants and transgenic strains have been published over the last 50 years. As a rhabditid nematode, *C. elegans* is considered a colonizer-type nematode displaying fast population booms in soil environments that are enriched with rotting fruit and vegetation, and eventual population busts are characterized by larvae moving into a non-proliferative hibernating form called dauer. In nature, *C. elegans* was found predominantly in temperate areas (Kiontke et al., 2011; Andersen et al., 2012) in different habitats ranging from wet shrublands to agricultural areas such as urban gardens or orchards (Frézal and Félix, 2015), where there is an abundance of decaying vegetation and rotting fruits. *C. elegans* was shown to associate with diverse bacteria in these environments that *C. elegans* can prey on but also bacteria that have a negative impact on the health of the nematode (Samuel et al., 2016).

To conduct experiments with *C. elegans*, practical protocols have been developed and used for decades to culture the nematode in the laboratory (Brenner, 1974; Stiernagle, 2006; Barrière and Félix, 2014). *C. elegans* is grown on the surface of an agar plate and fed with *Escherichia coli* bacteria as a food source. Although this method optimizes fast nematode growth for laboratory genetic experiments, it is different from the natural ecology of *C. elegans*. For instance, laboratory studies use a single stable bacterial population that is not naturally associated with *C. elegans* as opposed to the diverse and dynamic populations of microbes in natural settings (Samuel et al., 2016). In addition, *C. elegans* inhabits a complex 3D environment of rotting fruit, vegetation, and soil in nature, in contrast to the simple 2D agar environment in a standard laboratory culture. To simulate a 3D setting in the laboratory, we and others have designed habitats that allow *C. elegans* to live and move in three dimensions (Kwon et al., 2015; Lee et al., 2016a,b; Tahernia et al., 2020; Guisnet et al., 2021; Steel et al., 2021). Such studies have shown specific genetic requirements for survival and reproduction in the 3D environment that is dispensable in 2D (Lee et al., 2016a). However, these environments still rely on a single species of bacteria and do not account for conditions such as variable microbes, matrices, and climate found in nature.

A standard laboratory protocol to cultivate *C. elegans* and other nematodes in their native soil and fruit habitat would be useful in understanding how dynamic populations of nematode predators shape microbial diversity, and conversely, which microbial factors promote or hinder population growth of *C. elegans*. Incorporating *C. elegans* genetics with this type of method would begin to allow us to identify how ecology and the environment may have shaped the genome of *C. elegans* and confer evolutionary relevance to many functions and phenotypes studied for decades at the genetic level. In addition, such a protocol would be useful to examine the overall effects of chemicals, toxins, or climate change on a complex ecological system.

In this study, we developed a novel protocol that allows researchers to study *C. elegans* biology in its natural habitat but in a controlled laboratory environment. We established a mesocosm that we term soil-fruit-natural-habitat (SFNH) where we grew *C. elegans* in potted soil with a rotting apple as the decaying vegetation for the habitat. A regular day-night climate cycling was applied to the habitat. After adding the nematodes to the habitat, large population increases occurred over time, and we eventually observed population busts as well.

## Materials and equipment

### Methods

#### Soil-fruit-natural-habitat mesocosm construction

For this study, 13 cm top d × 11 cm bottom d × 11.8 cm ht. plastic gardening pots were purchased from a local horticulture store, and *Malus domestica* (Fuji apple) was purchased from a local supermarket or local fruit vendors to construct the SFNH. Apples bought were stored in a cold storage room at 4°C if not used immediately for the experiment. The storage period of the apple is a maximum of 3 weeks, and any fruits stored after that period were not used for the experiments and were discarded.

**Figure 1:**
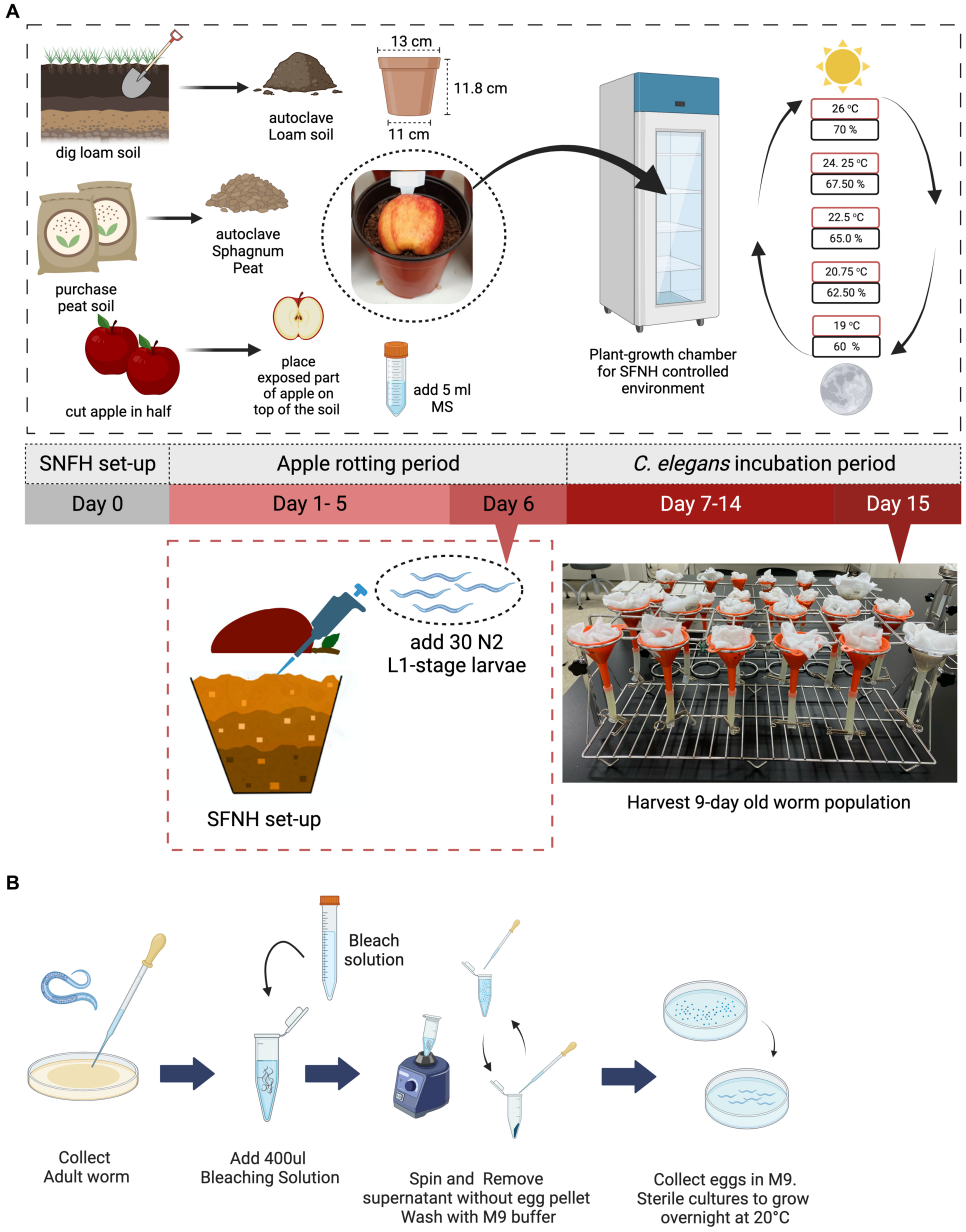
Setting up the soil-fruit-natural-habitat mesocosm. An SFNH mesocosm was set-up by adding autoclaved peat and loam soil-mix in a plastic pot. In total, 5 mL of microbial solution (MS) was added on top of the soil before the halved apple was put in place. The set-up is then placed inside the growth chamber where SFNH with *C. elegans* was incubated; shown are the temperature (red) and humidity (black) cycling parameters. After 6 days of the apple-rotting period, 30 L1-stage *C. elegans* larvae were added by lifting the rotten apple (some soils are attached) and pipetting M9 buffer containing the larvae on the surface of the soil. **(A)** 15 days experiment from setting-up SFNH (day 0) to harvest day (day 15) where the 9 days *C. elegans* population was harvested is conducted. **(B)** Sterile synchronization process of L1-stage *C. elegans* larvae. Created with BioRender.com.

Mesocosm construction was performed by adding a soil mixture consisting of a 50:50 peat and loam soil ratio (Figure 1A; materials and equipment used are presented in Tables 1 2). Peat soil from Sphagnum peat moss was purchased from a local horticultural supplier while the loam soil was obtained from a patch of land near the Greenhouse of Mirae Hall Building at Yonsei University in Wonju, Korea. The soil mixture was autoclaved for 15 min at 120°C (HYSC Autoclave, Seoul, South Korea) then cooled down before placing into the plastic pot. The pot was filled with the soil mixture to approximately 85% of its volume without compacting the soil. Afterward, 5 mL of pre-prepared microbial solution (see the preparation of microbial solution for SFNH) was added to the surface of the soil, ensuring that the whole surface was wetted with the solution (Figure 1A).

**Table 1:** Materials used for setting up SFNH including materials used for preparing the MS and harvesting *C. elegans*.

Material	Specifications	Protocol	Manufacturer
Apple	*Malus domestica*	SFNH	
Peat soil	H2-H4 on the von Post scale	SFNH	
Plastic garden/flower pot	13 cm top diameter 11.8 cm ht. 11 cm bottom diameter	SFNH	
Plastic funnel	50 mL	Baermann Funnel technique	
Silicon tube	Size 7	Baermann Funnel technique	Korea Ace Scientific Co., Seoul, South Korea
Steel metal clamps	12 mm	Baermann Funnel technique	EGLab, Incheon, South Korea
Universal funnel stand	12 holes; stainless steel	Baermann Funnel technique	SciLab^®^, Seoul, South Korea
Muslin cloth	60 × 60 cm	Baermann Funnel technique	
Conical tube	50 mL	Baermann Funnel technique	SPL, South Korea
Petri dish	60 mm × 15 mm	Counting worm aliquot	SPL, South Korea
Eppendorf tubes	1.5 mL	L1 synchronization	Axygen, South Korea
Petri dish	35 mm × 10 mm	L1 synchronization	SPL, South Korea
Serological pipette	5 mL		SPL, South Korea
Serological pipette aid			Neptune, South Korea
Micropipette	1,000 μL		
Micropipette tip	1,000 μL–1,250 μL		Neptune, South Korea
Micropipette tip	10 μL		ACE, South Korea
Weighing dish	Large		

**Table 2:** Equipment used for setting up SFNH including equipment used for preparing the MS and storage equipment.

Laboratory equipment	Specifications	Manufacturer/Distributor
Growth chamber (JSPC-300C)	Humidifier: Water Boiling Humidifier Temp. Range: +0°C–60°C (Light Off JSPC-300C) Illumination: 0–30,000 Lux by 8 Step Combination	JS Research Inc., Gonju, South Korea
HYSC Autoclave	Capacity: 100 L	Hanyang Science Lab Co. Ltd., South Korea
JSR Microcentrifuge	Max RCF: 1800 g RCF Capacity: 8 × 0.2, 0.5, 1.5, 2.0 mL tubes, 4 × 0.2 mL PCR Strips	JSR, South Korea
Stereo microscope	Zooming Range: 0.8–3.5×/0.7–3× Working distance: 100 mm	Nikon C-DS, Japan
Low temperature incubator	Heating: Forced air convection Temperature range: ambient +0°C to 70°C	JSR, South Korea
Convection incubator	Temperature range: ambient +5°C to 70°C	Samheung Instrument, South Korea
Centrifuge	Max. RPM: 5500 Max. RCF: 6230 Max. Capacity: 15 mL × 32	Hanil Science Co. Ltd., South Korea
Vortex	Speed Range: 0–3,300 rpm Shaking Motion: Orbital	Daihan Scientific Co Ltd., South Korea
Nihon Ultra-low Temperature Freezer	Temperature range: −70°C to −80°C Max temperature: −90°C	Nihon, Japan

Afterward, without prewashing to maintain the apple surface microbiome, the apple was cut vertically in half, and the exposed part was placed facing the soil surface (Figure 1A). Cutting the apple in half was performed to hasten the rotting of the apple. The surface of the apple was keenly observed for any signs of fungal contamination, especially contamination from inside of the apple by *Rhizopus stolonifer*. Any apple found contaminated was considered not usable for the experiment.

*C. elegans* added to the SFNH were developmentally synchronized, and 30 larvae-stage L1 worms were added. L1 is the first and earliest stage of the four larva stages in *C. elegans*. Synchronization of the worm (Figure 1B) followed the standard bleaching procedure (Stiernagle, 2006), where gravid adult worms were initially washed from a plate using S-basal buffer (5.85 g of NaCl, 1 g of K_2_HPO_4_, 6 g of KH_2_PO_4_, and 1 L of H_2_O) and transferred into 1.5 mL Eppendorf tubes and then left to settle for 2 min. The supernatant was discarded, and 400 μL of pre-prepared bleach solution (1 mL of stock bleach, 2.6 mL of autoclaved 3D H_2_O, and 0.4 mL of NaOH) was added per tube. The body of the gravid worms was then disintegrated, so the eggs could be released. Disintegration was performed for 5 min using a vortex with periodic pauses. After observation that the worms were completely disintegrated, the tubes were topped up to 1.5 mL with S-basal buffer and centrifuged for 2 min using a microcentrifuge (JSR microcentrifuge, Chungchungnam, South Korea). The supernatant was discarded, and the buffer was added and centrifuged again for two more times. The collected eggs were then resuspended in the buffer and transferred to a 35 mm plate (SPL, Gyeonggi, South Korea) and were placed at 20°C in a low-temperature incubator (JSR, South Korea) and left overnight. After the eggs hatched overnight, the buffer containing the worms was transferred to 1.5 mL Eppendorf tubes. An aliquot of the buffer was established to ensure that 30 L1 worms would be transferred to the soil. This was performed by pipetting an aliquot and counting the number of worms per aliquot under the microscope (Nikon C-DS, Japan). The amount of the aliquot varies from 5 to 20 mL depending on the number of worms hatched.

After a 6 days apple rotting period, the aliquot of 30 L1 worms was added to the soil surface (Figure 1A), and then, the set-up was placed in a plant growth chamber (JSPC-300C, JS Research Inc., Gongju, South Korea) with ramped control of temperature and humidity and illumination cycling to simulate day and night (Figure 1A; a detailed setting for the growth chamber is shown in Table 3). We chose 30 synchronized L1 worms as a result of some preliminary experiments showing that 30 was the smallest number that resulted in consistent and reproducible *C. elegans* population growth. Four pots were placed in each rack of the chamber—the total number of racks in the chamber is adjustable—and no rotation of the pots’ initial position was done throughout the experimental period. A mesh net was used to cover each pot to prevent any insects from contaminating the SFNH mesocosm with outside nematodes or microbes. The SFNH mesocosms were then incubated for 9 days to allow the growth and reproduction of the nematode. Each pot was watered every 2 days to make sure that the soil and apple did not dry up.

**Table 3:** The list of buffers and reagents that were used for setting up SFNH, MS preparation, and harvesting of *C. elegans*.

Media/Buffer	Preparation
NGM agar	5 g NaCl, 17 g Bacto agar, 2.5 g Peptone, 975 mL 3D H_2_O after cooling to 55°C add: 1 mL 1 M CaCl, 1 mL 1 M MgSO^4^, 1 mL 5 mg/mL Cholesterol in ethanol, 25 mL 1 M KPO_4_ buffer
S-basal buffer	5.85 g NaCl, 1 g K_2_HPO_4_, 6 g KH_2_PO_4_, 1 L H_2_O
Bleach solution	1 mL stock bleach, 2.6 mL autoclaved 3D H_2_O, 0.4 mL NaOH
0.85% NaCl buffer	0.85 g NaCl and 1 L dH2O
M9 buffer	3 g KH_2_PO_4_, 6 g Na_2_HPO_4_, 5 g NaCl, 1 L H_2_O, and 1 mL 1 M MgSO4 added after autoclaving

#### The preparation of microbial solution for SFNH

**Figure 2:**
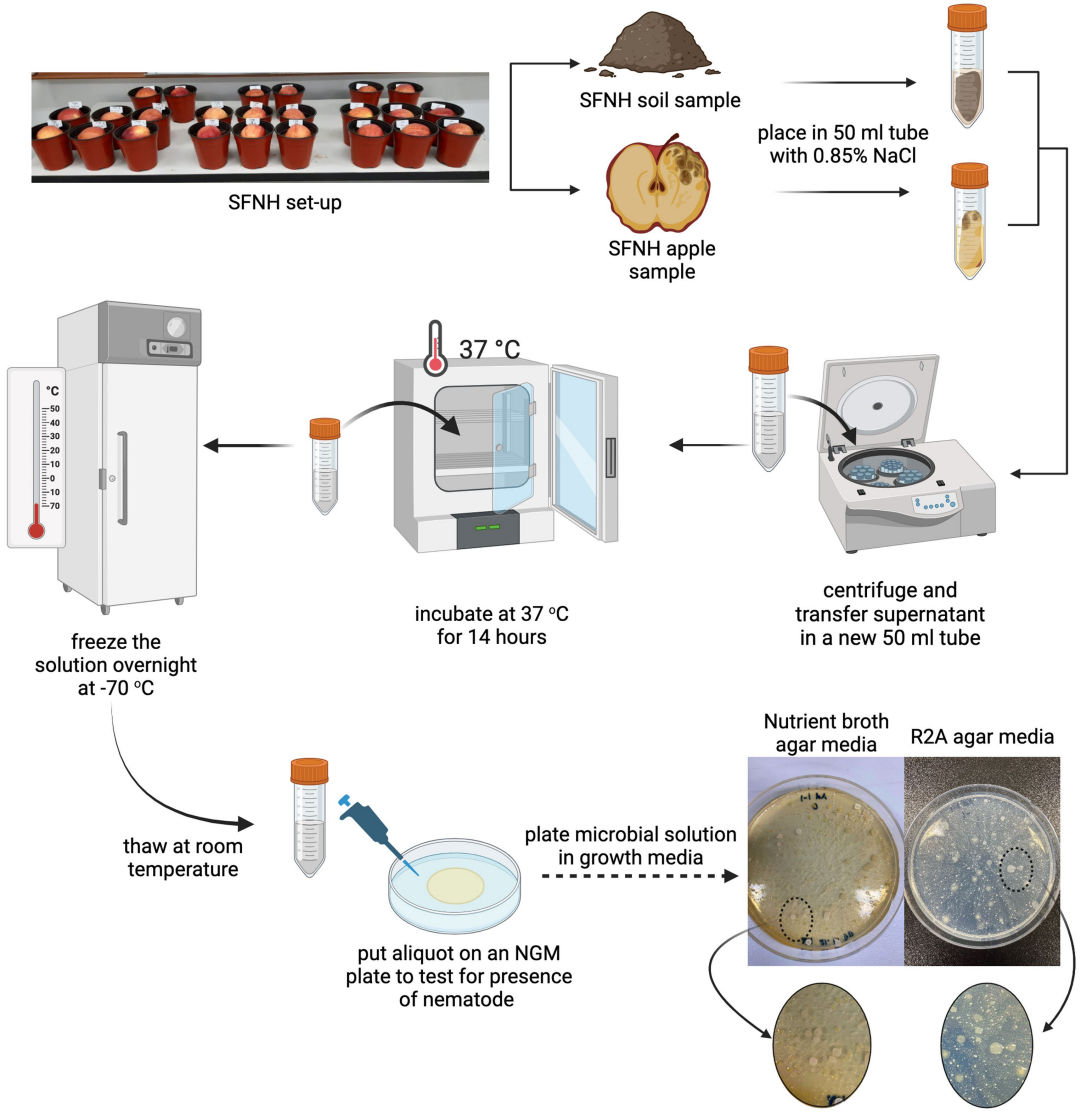
Microbial solution (MS) preparation. To replace the microbiome that was stripped off the soil due to autoclaving, we isolated the microbiome from a previous SFNH where *C. elegans* and other nematodes grew abundantly. (Top) The soil and apple samples from this mesocosm were placed into conical tubes with 0.85% NaCl separately. (Middle) The mixture was centrifuged, and the supernatant was used as the microbial solution (MS). To make sure that all nematodes, insects, and eggs from the apple and soil samples were killed, the MS was incubated at 37°C for 14 h, frozen overnight at −70°C, and then thawed the next day. (Bottom) Afterward, an aliquot of the MS was plated in NGM agar plates to check for any nematode growth. Upon clearance, the solution was also plated in nutrient broth, R2A, and PDA agar plates to confirm the presence of microorganisms. Created with BioRender.com.

One of our earlier SFNH experiments that was performed on an open benchtop rather than inside a climate-controlled incubator resulted in SFNH pots remarkably rich in nematode growth. The exposed pots were observed to be swarmed by fruit flies, a vector for nematode dispersal (Lee et al., 2012), and nematodes found in the pots were not only *C. elegans* but also other nematodes. We, however, surmised that the apples and soils in these samples contained microbial bonanzas that would contribute to rich nematode growth. Therefore, from these pots, 10 g of apple and soil samples were collected and placed in 50 mL Falcon tubes separately (Figure 2). The NaCl buffer (0.85%) was added to the tubes until it was filled to the brim. The tubes were then vortexed for 3 min and centrifuged (Hanil Fleta 5, Gimpo, South Korea) for 5 min at 3,000 rpm at 20°C. The supernatants were transferred to new 50 mL tubes and once again centrifuged for 5 min at 3,000 rpm. The new supernatants were then kept in a convection incubator (Samheung Instrument, Gangnam, South Korea) for 14 h at 37°C to kill any nematodes and eggs that might have been collected during the sampling. This process was performed specifically to get rid of any nematode larvae and eggs that might contaminate future SFNH mesocosm. After incubation, the collected MS was stored at −70°C (Nihon Ultra-low Temperature Freezer, Gyeonggi, South Korea) overnight. The solutions were then thawed the next day, and an aliquot of each sample was placed in a nematode growth media (NGM) plate with *E. coli* to check whether any nematodes were present in the stock MS. Once cleared, the stock MS was again stored at −70°C for further experiments. The survival of the microorganisms was also checked by plating 100 μL of serially diluted on the growth agar media. Figure 2 shows bacterial growth from MS solutions diluted to 1:10.

#### The harvesting protocol for worm population analysis

**Figure 3:**
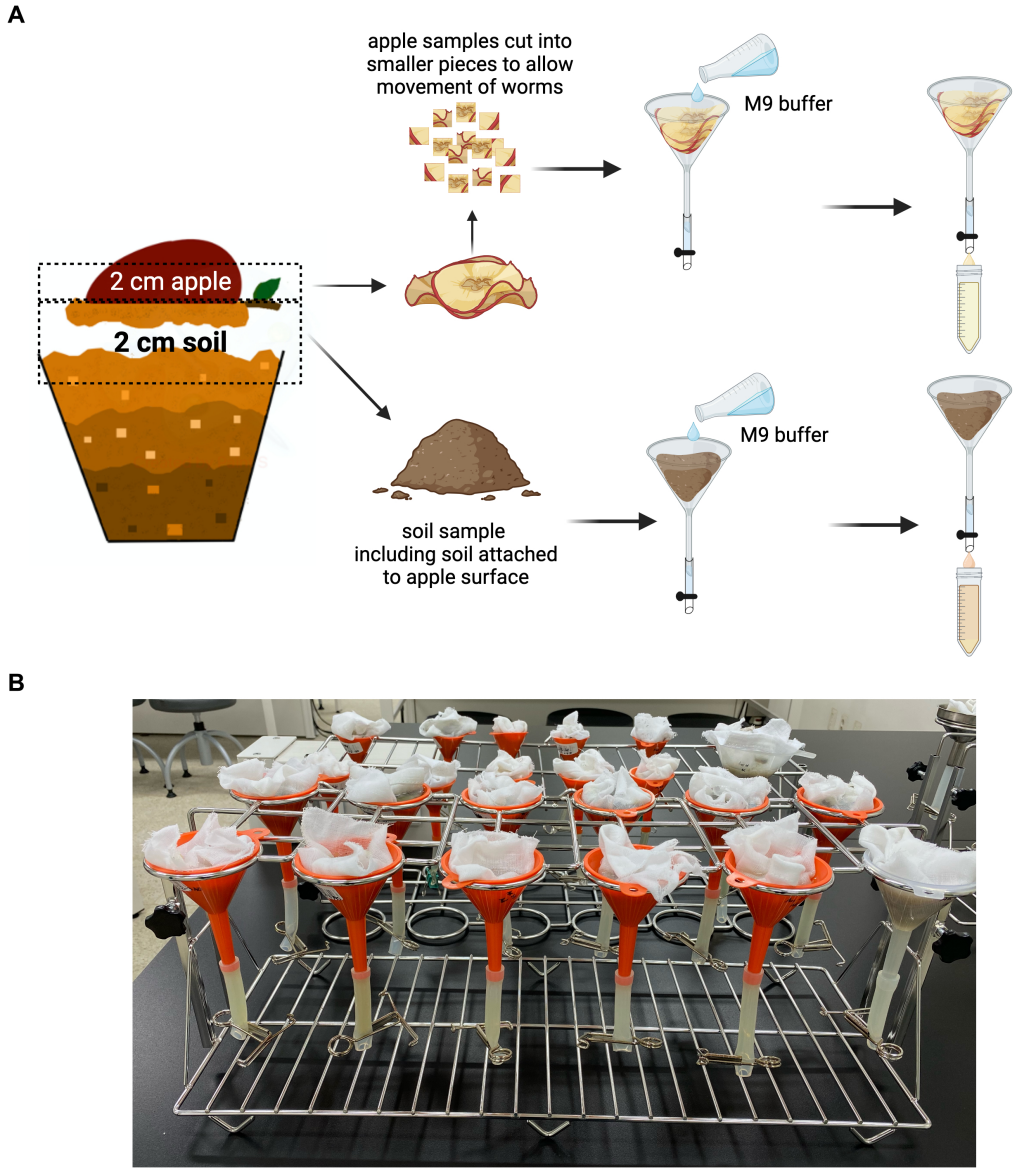
Harvesting of *C. elegans* from the SFNH mesocosm. **(A)** Baermann funnel technique was used to isolate the worms from both the soil and the apple. Muslin cloth was used to line the inner surface of the funnel, and then, the apple and/or soil was placed on it. M9 buffer was poured afterward, and the set-up was let to stand for 4 h to give time for the worms to swim to the clamped silicon tubes. The buffer was then collected into 50 mL conical tubes and used for worm population analysis. **(B)** The funnels are placed on a funnel-holder rack while letting it stand for 4 h. Created with BioRender.com.

The worm population was harvested using a modified Baermann Funnel technique (Figure 3A). Plastic funnels (50 mL) were purchased from a local hardware store. The funnels were fitted with size 7 silicon tubes (Korea Ace Scientific Co., Seoul, South Korea), and the tubes were closed using 12 mm stainless steel metal clamps (EGLab, Incheon, South Korea). The funnels were then suspended on a 12-hole stainless steel universal funnel stand (SciLab^®^, Seoul, South Korea) and lined with a muslin cloth (Figure 3B).

Harvesting of apple and soil samples was performed separately. From the exposed surface of the apple, a 2 cm tissue sample was cut, placed on the muslin cloth on the funnel, and 50 mL of M9 buffer was added (buffers and buffer recipe used are presented in Table 4). Approximately 2 or 3 cm (from the surface) of soil mixture was placed in the muslin cloth, and then, 50 mL of M9 buffer was added; the soil attached to the exposed surface of the apple was removed and added to the soil sample. A pre-wetting procedure, using the same buffer, may be performed on the muslin cloth to ensure that the cloth does not absorb too much of the 50 mL buffer added to the sample. This ensures that there is enough buffer to permit the worms to move around and swim through the samples. The set-up was incubated for 4 h to allow the worms to swim downward and settle on the bottom of the silicon tube. The worms were then recovered by removing the metal clamps from the silicon tubes and collecting the buffer in a 50 mL conical tube.

**Table 4:** Temperature, humidity, and light cycling inside the plant growth chamber where SFNH was incubated.

Parameter	Value	No. of hours
Temperature (°C)	26.00	6
	24.25	4
	22.50	4
	20.75	4
	19.00	6
Humidity (% RH)	70.00	6
	67.50	4
	65.50	4
	62.50	4
	60.50	6
Illumination (lux)	17,600–17,900	6
	15,300–15,600	4
	12,000–12,400	4
	7,200–7,300	4
	0	6

#### A comparison of developmental stage in *C. elegans* grown in OP50 plate and SFNH

To compare growth and development in SFNH-grown *C. elegans* with that of the standard laboratory culture methods, experiments with nematode growth media agar plates containing a lawn of OP50-strain *E. coli* bacteria were performed. Two experiments with OP50 NGM plates were designed. The first started with 1 young adult (YA) on OP50 NGM plates while the second started with 30 L1 larvae worms. In this second experiment, a styrofoam half-sphere, similar to the “fake apple” used in the SFNH experiments, was placed on top of the Petri dish cover to simulate the apple in SFNH. The plates were then placed inside the growth chamber with the exact temperature, humidity, and light conditions used for SFNH and incubated for 5 days. Worms were then counted every 24 h for 120 h.

## Results

### The simulation of *C. elegans* environment in the soil-fruit-natural-habitat

Our goal is to design a protocol that can simulate natural dynamic fluctuations in *C. elegans* and microbial populations in a soil environment enriched with decaying organic matter. A previous study observed that large populations of mature *C. elegans* were found in moderately rotten apples that fell to the ground under trees (Samuel et al., 2016). We decided to base our SFNH around this rotting apple and soil model by inducing apple rot from autoclaved soils enriched with microbes and then adding *C. elegans* to the soil underneath the apple to monitor *C. elegans* population growth over time. In nature, *C. elegans* populations were observed in spring and fall seasons in temperate environments, such as in France and Germany, and generally found in more humid areas (Félix and Duveau, 2012; Petersen et al., 2014). *C. elegans* also grows and reproduces well at temperatures between 15°C and 25°C. To simulate these optimal natural conditions for *C. elegans* growth with daily day/night cycling, we set our incubator “climate” to cycle between 19°C and 26°C, 60–70% humidity, and 12 h light/dark cycles every 24 h (Figure 1A) for the SFNH mesocosm. In addition, we conducted experiments to optimize several more factors for *C. elegans* growth and reproduction: soil composition, microbial solution concentration, and apple rotting period.

### Soil composition affects nematode population growth in the SFNH

**Figure 4:**
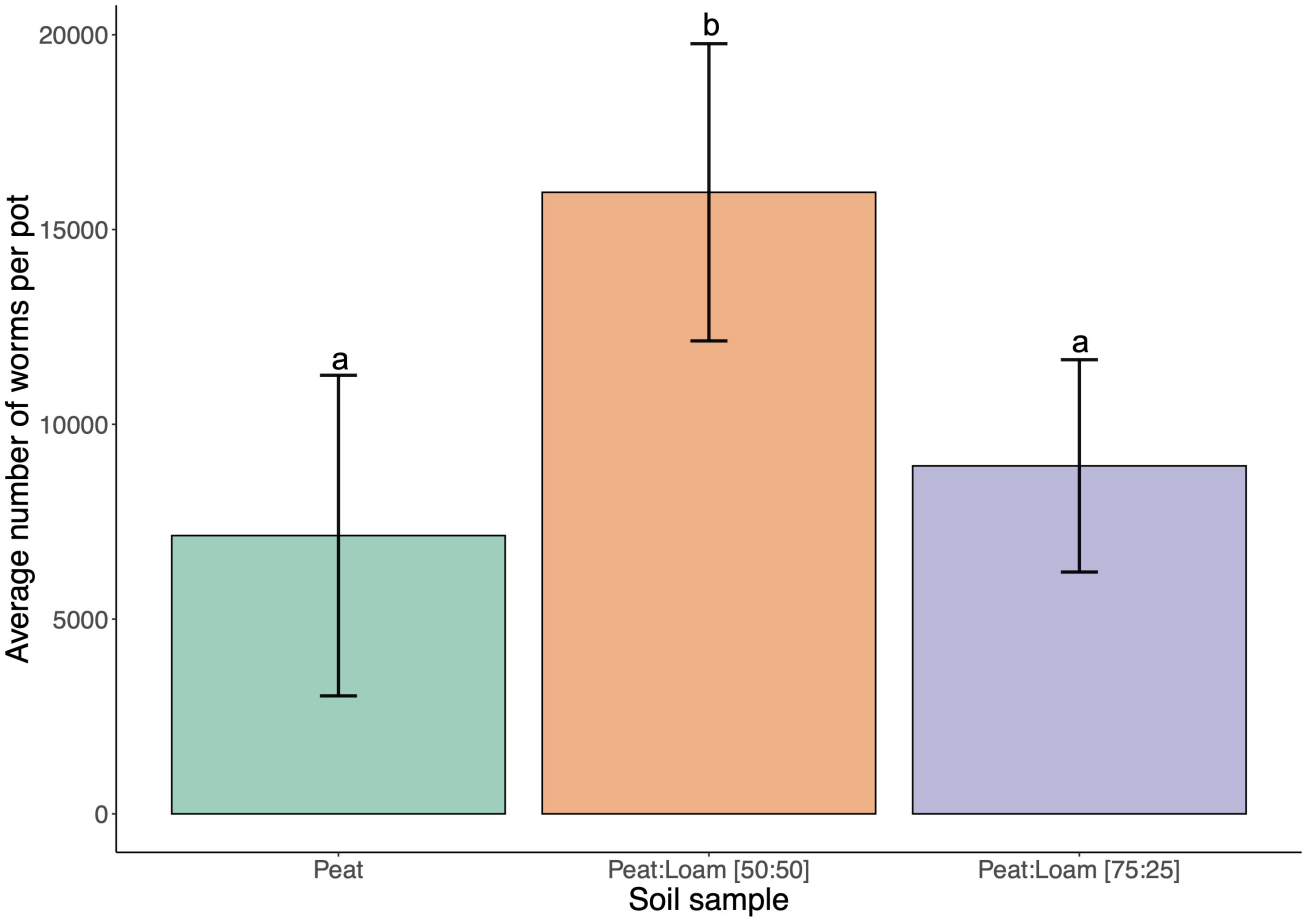
*C. elegans* population growth is affected by soil composition. The results of SFNH mesocosm experiment where worms were incubated for 9 days. Reproductive fitness of the worm was observed highest when the soil is a mixture of 50% highly organic peat and 50% loam soil. Different letters denote differences in the mean using One-way ANOVA (*p*-value < 0.5) and Duncan’s Multiple Range Test (DMRT; alpha value = 0.5). Error bars are standard deviations (*n* = 3).

Soil type and composition can have a large effect on microbial populations and diversity (Lombard et al., 2011; Schreiter et al., 2014). *C. elegans* was previously found in fruit orchards (Frézal and Félix, 2015; Samuel et al., 2016) and agricultural settings that are associated with more peat or loamy soils rather than sandy or silt soils. We first conducted preliminary experiments with 100% peat. However, we found that peat soils have low water retention causing dryness in the SFNH. We also noted that peat contains high organic materials, and this property may have a selective effect on the microbial growth in the substrate. For these reasons, loam soil was added to peat soil. Indeed, after 9 days of incubation in the SFNH, *C. elegans* populations increased by 25% in 75:25 peat to loam soil and by 123% in 50:50 peat to loam soil (Figure 4). Thus, we found a mixture of 50:50 peat to loam soil allowed for higher *C. elegans* population growth.

### Microbial solution concentration affects *C. elegans* population growth

Previously, wild *C. elegans* obtained from rotting apples was associated with an abundance of diverse bacteria that were positively correlated with worm growth (Samuel et al., 2016). To obtain a soil microbiome that could stimulate *C. elegans* growth, we washed a large soil sample that was found rich in nematode populations and used this microbial wash solution as a standard microbial solution (MS). This MS is added to the SFNH to ensure that each ecological succession experiment commences with a uniform and consistent microbial population. In the process of establishing MS, we subjected the solution to 37°C incubation to make sure that no nematode larvae or egg would survive in the solution since the array of nematodes found from the source SFNH set-up consisted of other nematodes alongside *C. elegans*. It is important to note that the process can alter the original microbiome from the soil-apple mesocosm. Therefore, this incubation process may be bypassed for experimenters who opt to use other microbial sources such as CeMBio (Dirksen et al., 2020).

**Figure 5:**
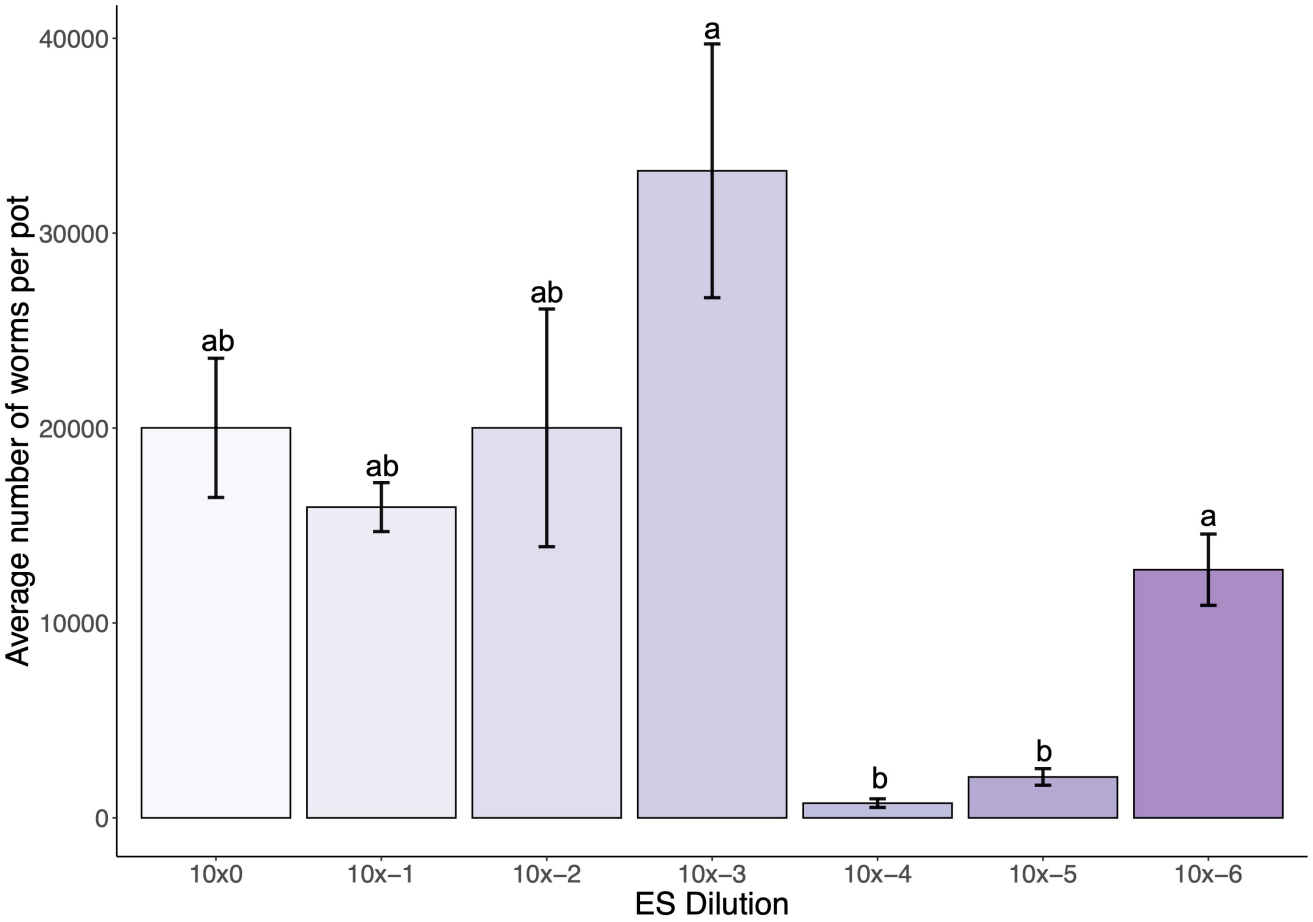
Dilution of microbial solution affects *C. elegans* population growth in SFNH mesocosm. In total, 5 mL dilutions of the original MS were added at starting of each SFNH experiment, and *C. elegans* populations were counted 9 days after adding the worms. Different letters denote differences in the mean using one-way ANOVA (*p*-value < 0.05) and DMRT (alpha value = 0.05). Error bars are standard deviations (*n* = 3).

To determine an optimal starting MS concentration in the SFNH mesocosm that would stimulate *C. elegans* population growth, we added various concentrations of the MS to the SFNH, added *C. elegans* larvae after 6 days, and counted the number of *C. elegans* present in each SFNH after 15 days. Interestingly, we observed high *C. elegans* populations of over 30,000 at an MS dilution of 1:1,000 and then a steep drop-off of *C. elegans* growth at a dilution of 1:10,000 (Figure 5). Thus, we determined that an MS dilution of 1:1,000 was optimal for *C. elegans* growth in the SFNH.

### The role of apple and the effect of the apple rotting period on *C. elegans* population growth

**Figure 6:**
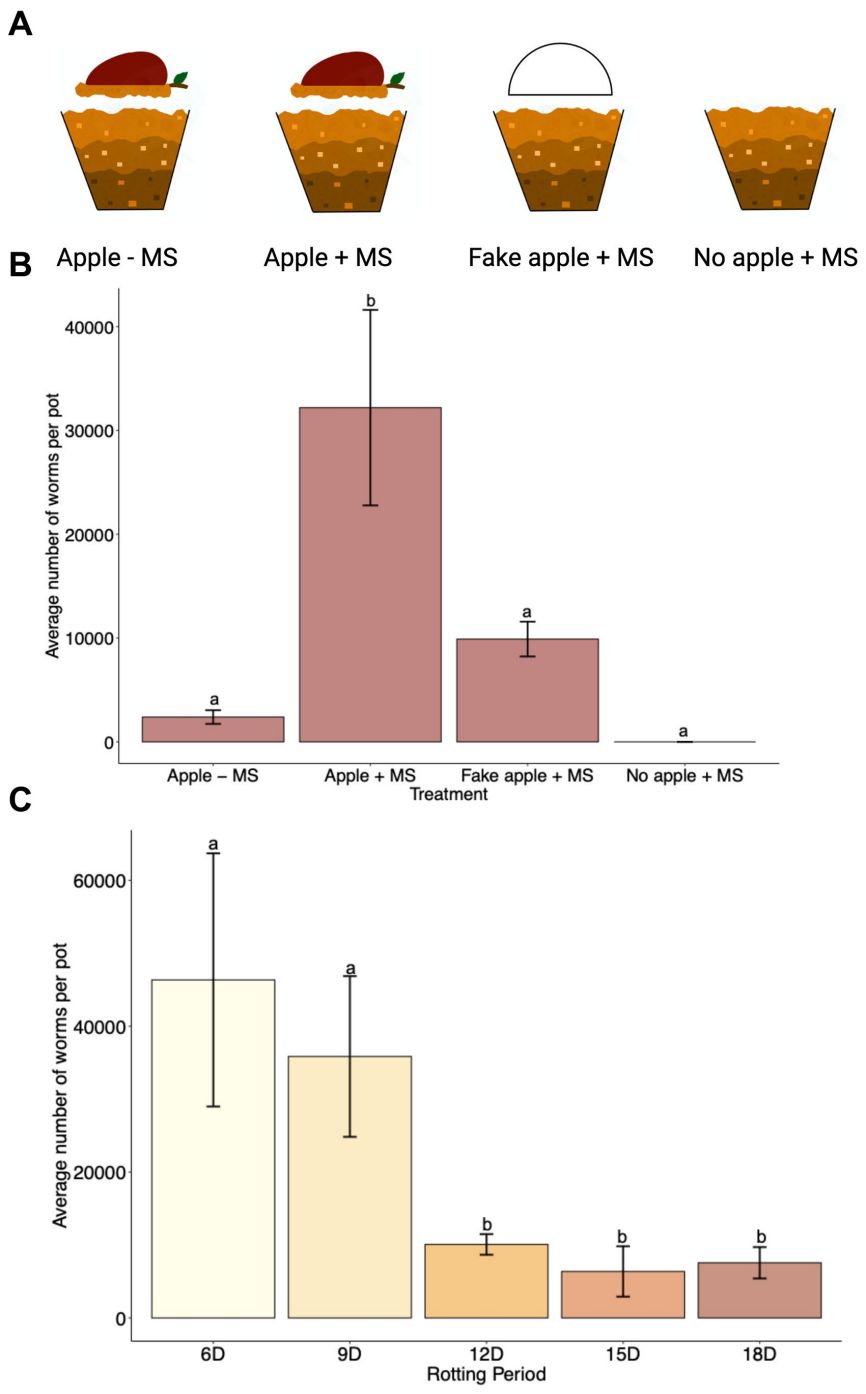
A combination of rotting apple and MS in the SFNH mesocosm provides greater *C. elegans* population growth. **(A)** For the experiment to analyze the significance of the apple in the SFNH mesocosm, four treatments were set up where either the apple, MS, or both were removed to observe the role of the apple in the mesocosm. **(B)** Data shows that the combination of rotting apple and MS allows for higher worm growth. *C. elegans* populations were counted 9 days after adding the worms. This figure also shows that worm growth, albeit much lower, was observed even when apple was not present in the mesocosm. Different letters denote differences of the mean using one-way ANOVA (*p*-value > 0.5) and DMRT (alpha value = 0.5). Error bars are standard deviations (*n* = 9). **(C)** Based on the results, 6 days apple rotting is enough to support *C. elegans* growth and reproduction in the SFNH mesocosm. *C. elegans* populations were counted 9 days after adding the worms. Different letters denote differences of the mean using one-way ANOVA (*p*-value < 0.5) and DMRT (alpha value = 0.5). Error bars are standard deviations (*n* = 3).

Several studies have confirmed the correlation between rotting apples and the presence of *C. elegans* in nature (Barrière and Félix, 2007; Petersen et al., 2014; Samuel et al., 2016). However, the presence of *C. elegans* in nature was also correlated with other fruits and vegetables, compost, and snails (Barrière and Félix, 2007; Petersen et al., 2014). We wanted to confirm whether the rotting apple itself rather than the presence of any object over the soil could contribute to *C. elegans* population growth. Therefore, we used the SFNH for a 15 days cultivation period under four different treatments (Figure 6A). We first tested whether the apple alone without exogenous MS treatment supported *C. elegans* growth. Although some worms were present after 15 days, large population growth was not supported with only the apple (Figure 6B). Next, we added MS together with the apple and found that this treatment supported large *C. elegans* population growth. We also hypothesized that the apple could itself act only as a protective material against soil desiccation in the nematode habitat. We tested this by replacing the apple with a half-apple-shaped styrofoam to simulate a “fake apple.” The “fake apple” does not provide any microorganisms or sugar sources for other existing microbes from the MS, but it does provide protection from direct heat, light, or water evaporation of the soil. Interestingly, some nematode growth was supported by the fake apple (Figure 6B), suggesting that the MS itself can provide bacterial growth for nematode populations to a limited extent. Finally, when either a real or fake apple is present, the MS itself cannot support any nematode growth, demonstrating the importance of maintaining a shaded and humid soil environment. Overall, the rotting apple together with the MS supports large *C. elegans* population growth.

It was previously reported that large proliferating populations of *C. elegans* were observed in nature trending toward highly decayed apples rather than lightly decayed apples albeit with a high degree of variability (Samuel et al., 2016). We sought to evaluate the period of rotting necessary to induce *C. elegans* population growth. After starting the SFNH experiments, we allowed the apples to decay for 6, 9, 12, 15, or 18 days before adding *C. elegans* larvae. Interestingly, 6 days of apple rotting provided the optimal environment for *C. elegans* growth in the SFNH (Figure 6C).

### Ecological succession assay using SFNH mesocosm shows a pattern of nematode growth that can mimic the natural environment

In nature or agricultural soils, colonizer-type soil nematodes, such as *C. elegans*, take quick advantage of flourishing bacterial populations in soils enriched by organic matter (Bongers and Bongers, 1998; Ferris and Bongers, 2006). Colonizer-type nematodes are characterized by rapid development, fast reproduction, and large brood sizes, resulting in booms of the population that follow with the growing bacterial populations (Bongers, 1990; Bongers and Bongers, 1998). In nature, large *C. elegans* populations are observed together with soils enriched with organic matter, such as rotting fruits, but an ecological succession in which growing nematode populations are tracked over time has not been conducted previously.

**Figure 7:**
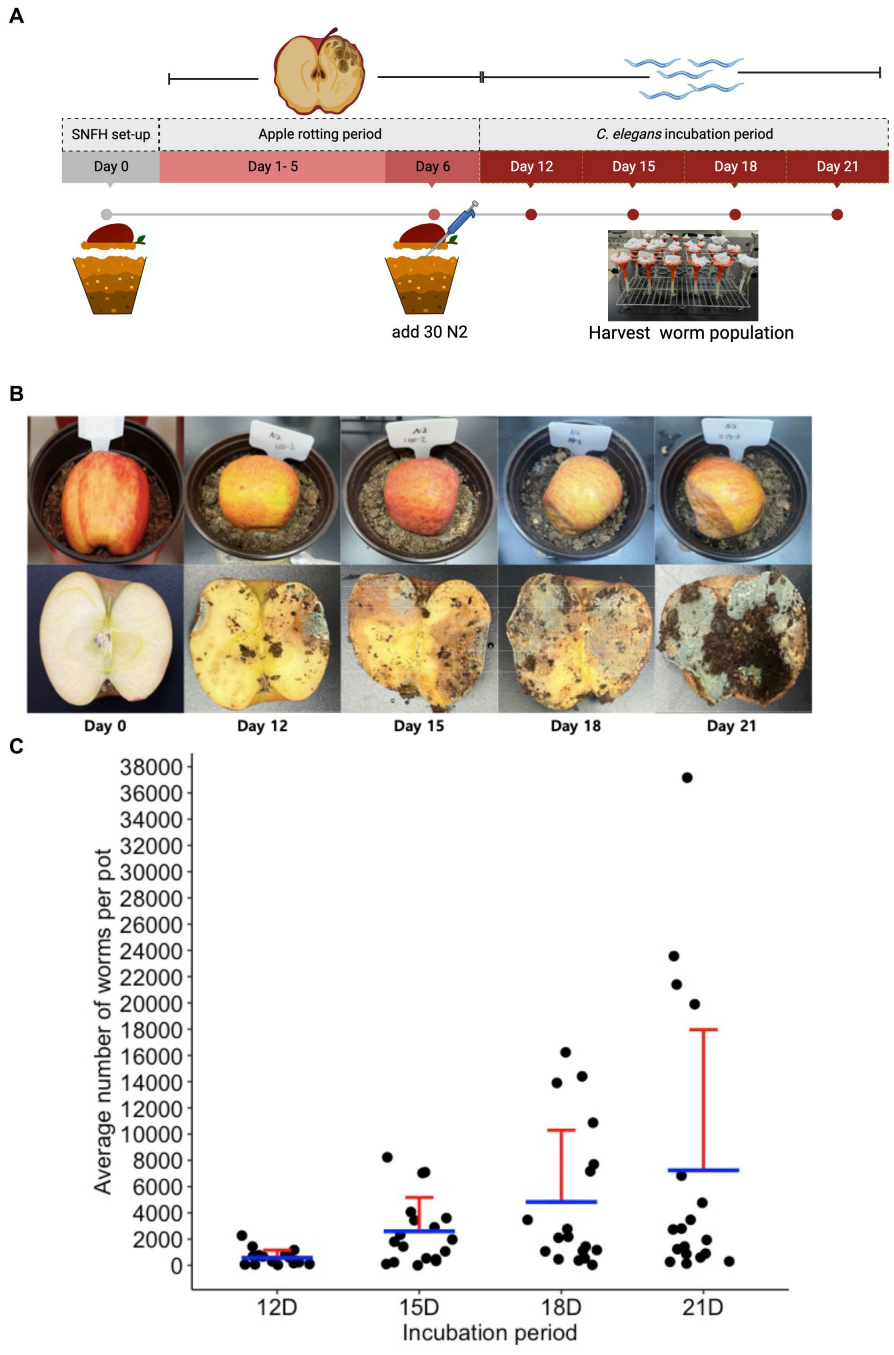
Ecological succession assay in the SFNH mesocosm. **(A)** Timeline for ecological succession (ES) assay where worm population was harvested at an increment of 3 days of growth. **(B)** Apple rotting in SFNH from the day of set up (Day 0) to harvest day (Day 21). **(C)** An increase in the *C. elegans* population was observed on day 15 of the ES assay, and a subsequent decline in the average population was observed from day 18 onwards. It was also important to note that some pots were observed (21D) to have more worms than the rest of the replicates for the same day treatment. Each pot is an individual ecological environment on its own and microenvironmental factors surrounding each pot might have caused this high number of worm population. Solid sphere = 1 pot, *n* = 18 pots for each day. Red lines = upper standard deviation; blue horizontal line = mean.

To observe if we can also notice the same ecology between the nematode population dynamics and the microbial population, we performed an ecological succession (ES) assay using the SFNH. Since we determined optimal conditions for the SFNH, we started SFNH pots with 50:50 peat to loam soil, with a half apple and an MS dilution of 1:1,000. After 6 days of apple decay, we added 30 L1-stage *C. elegans* larvae and allowed the worms to grow and populate the SFNH for 6, 9, 12, or 15 days. Thus, SFNH pots were incubated for 12, 15, 18, and 21 days (Figures 7A,B).

To assess the ecological succession of *C. elegans*, we measured the average worm population in the pots for each day and initially observed increasing populations from day 12 to day 21 of the experiment (Figure 7C). It was also noted that the worm population showed increased variation over time, which was similar to the results by Petersen et al. (2023). With such large variations in populations, we wondered whether the nematodes could disperse to lower areas around the pot. We found that few worms accessed a lower layer of soil from 12 days to 18 days, whereas at 21 days, some of the pots had hundreds and even over a thousand worms at the lower layer (Supplementary Figure S1). This shows that over time, the worms will occasionally disperse to further areas of the mesocosm. However, the reason for this variation requires further analysis.

### Comparison of *C. elegans* population growth and developmental stages between standard plate culture and SFNH mesocosm

**Figure 8:**
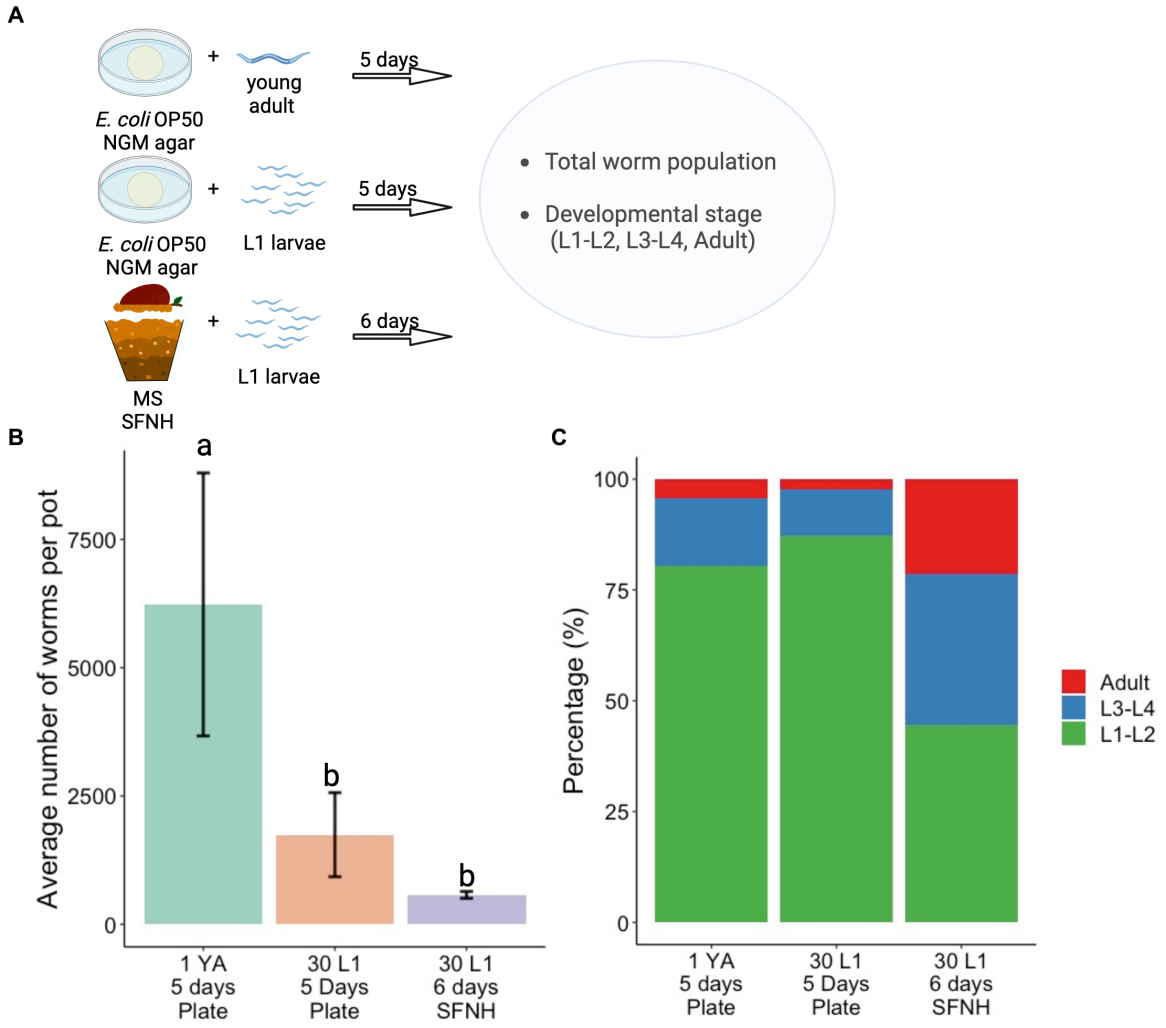
Comparison of developmental stages in plate and SFNH grown *C. elegans*. **(A)** Illustration of the protocol performed to compare the growth and development of *C. elegans* in SFNH versus the traditional OP50 plate. **(B)** Comparison of the average number of worm per pot among plate-grown and SFNH-grown *C. elegans*. **(C)** Percentages of different worm stages across the experimental set-ups show that similar to plate cultivation, SFNH cultivation also results in a higher L1 larvae percentage compared with L3–L4 and adult stages. YA, young adult; L1, L1 larvae; the number before letters indicates the number of worm(s) initially added, and the number of days indicates the cultivation period. Different letters denote differences in the mean using one-way ANOVA (*p*-value < 0.01) and Tukey’s HSD (alpha value = 0.05). Error bars are standard deviations (*n* = 3). Created with BioRender.com.

Conventionally, *C. elegans* are grown on NGM plates with *Escherichia coli* OP50 as feed in the laboratory. To compare nematode growth and development between the standard plate culture and SFNH, we assessed population growth and development on NGM plates for 5 days and compared these with SFNH nematode population growth and development incubated for 6 days (Figure 8A). We performed two 5 days experiments on NGM plates: one experiment started with 1 young adult (YA) worm to demonstrate the high rate of reproduction of single adult *C. elegans* during optimal laboratory culture and another experiment was more similar to the SFNH experiment starting with 30 L1 larvae. *E. coli* food was plentiful for the entire 5 days experiments. However, we were unable to proceed to 6 days experiments because the large populations of worms had completely consumed the food, resulting in suboptimal starvation conditions.

As expected, population growth skyrocketed well into the thousands starting with 1 YA, whereas the growth was similarly slower when starting with 30 L1 larvae on either plate or SFNH (Figure 8B and Supplementary Tables S1, S2). This is mostly due to the extra days required for the L1 larvae to grow into reproducing adults. Although the 6 days population of SFNH was less than half of the 5 days plate population starting with 30 L1 larvae, the difference was not statistically significant, demonstrating similar growth in a 5 days plate and 6 days SFNH (Figure 8B). However, after 5 days, a large majority of the worms on either plate experiment were young L1–L2 larvae in contrast to SFNH, where a large percentage of the worms were adults or L3–L4 larvae (Figure 8C). Taken together, these experiments demonstrate that reproduction and possibly development may be slightly slower in SFNH conditions than in optimal laboratory conditions on NGM plates with *E. coli* bacteria.

## Discussion

This study presents a novel protocol that enables studying *C. elegans* biology in a semi-controlled “natural environment”—the SFNH mesocosm. The growing interest in studying *C. elegans* biology in its natural environment has led to numerous studies that repeatedly isolated *C. elegans* from its natural habitat to characterize the nematode’s biology and ecology from worm population (Petersen et al., 2014) and life cycle (Félix and Duveau, 2012) to microorganism associated with the worm (Samuel et al., 2016). All these studies were conducted by sampling *C. elegans* from nature, where environmental conditions are not controlled, and data on macroenvironmental and microenvironmental parameters are scarce or unknown. The protocol we present here allows the study of *C. elegans* biology in an environment that simulates its natural habitat but is semi-controlled and can be carried out throughout the year in the confines of the laboratory. This SFNH mesocosm allows for controlling environmental parameters such as temperature, humidity, and light cycle and can be used to test *C. elegans* behavior to environmental changes that can help identify triggers to certain genes expressed that cannot be done using the conventional plate assay. All the factors making up the mesocosm were tested to ensure each component (soil, apple, and MS) is optimal for *C. elegans* growth and development, and the mesocosm itself was used for an ecological assay to show that it can simulate the growth of the nematode in its natural habitat.

In setting up the SNFH mesocosm, we used two types of soil—peat and loam soil. “Peat soil” from *Sphagnum* peat moss (H2–H4 on the von Post scale) is characterized by low pH and bulk density, high porosity, high electrical conductivity (EC), and high water and air capacity (Schmilewski, 2008; Pot et al., 2022). “Peat soil” was chosen since peat is free of pests and pathogens (Schmilewski, 2008) and is thus pesticide-free. Loam soil, on the other hand, which is composed of sand, silt, and clay particles is characterized by good water and nutrient retention (Bennett, 2015). Our test on soil composition showed that a 50:50 ratio of peat and loam soil allows for *C. elegans* population growth that is 123% higher than worm growth in peat soil alone. Since *C. elegans* has been reported to proliferate better in enriched soil with high microbial growth (Bongers and Bongers, 1998; Félix and Duveau, 2012), one possible reason to explain this higher nematode population is the effect of soil type on the microbial community in the soil that can, in turn, affect *C. elegans* food sources. According to studies, soil type, matrix, and physicochemical properties have a pronounced influence on the dynamics of microbial community structure (Lombard et al., 2011; Schreiter et al., 2014). Soil is a highly structured environment in which the degree of structure depends on its composition. Its complex structure is composed of micro-aggregates (mainly organic matter and clay) and macro-aggregates (several micro-aggregates lined together), where the microbial distribution is not even (Lombard et al., 2011). Microscale studies on soil illustrated localized peaks and lows of bacterial density (Grundmann et al., 2001; Nunan et al., 2002; Dechesne et al., 2003; Gonod et al., 2003; Nunan et al., 2003). These microbial community distributions are reported to be linked to local conditions inside the soil matrix, where soil moisture and pH were shown to affect the microbial community composition (Lombard et al., 2011). The moisture content of the soil, for example, can cause selective microbial growth in the soil (Brockett et al., 2012), while the variation of microbial community structure over a field was, to a large extent, due to soil pH (Fierer and Jackson, 2006; Baker et al., 2009; Rousk et al., 2010; Bru et al., 2011). In our tests, we did not use pure loam soil because originally *C. elegans* are found in areas where soils are rich in organic matter (Lombard et al., 2011; Petersen et al., 2014; Frézal and Félix, 2015), and we aimed to mimic these conditions. In addition, we found that without added peat soil, 100% loam is highly compacted, making nematode recovery difficult. In line with this, the importance of the porosity and organic matter of the loam soil should be assessed by future research, especially in the case of pure loam soil.

Originally, *C. elegans* (mostly dauer) was isolated in rich soil or compost (Hodgkin and Doniach, 1997); however, recently, feeding and reproducing stages have been found in decomposing plant material (Félix and Duveau, 2012). Rotting substrates, especially sugar-rich with high water activity plant materials, harness high bacterial population and diversity (Frézal and Félix, 2015; Lievens et al., 2015). Therefore, these habitats provide ample food sources for *C. elegans*. In highly rotten apples, for example, *C. elegans* was found to have a higher population than in less rotten ones (Samuel et al., 2016). Because of these reports and the fact that apple is available in Korea all year long, we selected apple (*Malus domestica*) as our “plant material.” However, our assay using SFNH mesocosm showed that the role of apple in *C. elegans* natural habitat does not end as merely a source of bacterial feed for the nematode. We also established that the apple, to an extent, provided protection to *C. elegans* by acting as a shade. The “fake apple” did not provide any sugar source to the overall habitat, therefore it cannot provide any carbohydrates for the microorganisms to feed on, which did not help support bacterial population growth. Instead, we surmise that it acted as a structure that prevents soil desiccation and direct exposure to light and high temperature. Moisture or humidity can affect *C. elegans* growth and behavior, and it has been observed that nematodes tend to associate themselves with animals that provide a more humid environment than most spiders or insects (Petersen et al., 2015). More importantly, moisture is imperative for the worm’s survival since most of the nematode’s life stages are sensitive to desiccation (Erkut et al., 2011). Light exposure was also found to influence *C. elegans* lifespan. According to De Magalhaes Filho et al. (2018), the length of light exposure is inversely correlated with nematode longevity. Therefore, the presence of the apple in our SFNH set-up is imperative for more than just one role.

Since we designed a mesocosm with apple as plant material, it is important to discuss a few challenges that we encountered and deemed important to note for future use of the set-up. From our experience, fungal contamination posed as the biggest hurdle during the winter months when freshly harvested apples are unavailable. Apples are reported to be susceptible to fungal contamination at pre-harvest, harvest, and post-harvest stages (Patriarca, 2019), but the most severe fungal diseases occur in post-harvest stages. During our experiment, we encountered what appears to be a combination of apple moldy core and core rot (Gao et al., 2013), where we observed black to gray fungus growth over the seed and carpel walls, the entire central portion of the core filled with mycelium, and white mycelia growing in the core region alongside wet core rot. According to Gao et al. (2013), *Alternaria*, *Cladosporium*, and *Trichothecium* are the main fungal genera causing these cases of “moldy core” in Fuji apples. Unfortunately, post-harvest fungal contamination is not easy to avoid without the use of fungicides. Since we do not want to alter any microbial community (soil and apple), we simply do an ocular inspection of the apples (skin and core after it was halved), to avoid using those that seem contaminated.

We also observed that the MS and MS concentration added to the mesocosm have a significant effect on the *C. elegans* growth. Data from the experiment determining the role of the apple also showed that the MS we are using is stable enough to allow *C. elegans* growth, where, despite adding MS with only a “fake apple,” we still observe the growth of the worm in the SFNH mesocosm. The MS concentration of 1:1,000 dilution that we ultimately used for our ES experiments was also established to be the optimum concentration of our MS stock that allowed for high *C. elegans* growth. We inferred that this could be explained by a possible decrease in microbial colony diversity and richness as the microbial solution is diluted (Franklin et al., 2001). The microbial community that is left after dilution could be fast proliferating but small enough to serve as feed for *C. elegans* and/or beneficial as more than feed. Interestingly, further dilution of the MS did not result in further worm population growth. In fact, dilution at 1:10,000 resulted in a steep decrease in the *C. elegans* population. This could be due to a further decrease in microbial population diversity, where only non-beneficial microbes might have been left in the highly diluted MS. However, to prove these hypotheses we need further analysis of the microbial profile and more assay of the microbes’ effect on *C. elegans* and *vice-versa*. In addition, our choice to create a microbial solution by washing the microbiome of the soil and apple rather than using CeMbio strains (Dirksen et al., 2020) was due to our goal to emulate variables as true to the natural habitat as possible. However, even though our established MS was enough to grow a bacterial population that drove *C. elegans* growth, it should be noted that the incubation and freezing process that was performed during the establishment of the MS may alter the original microbiome from the mesocosm. Thus, future experiments that opt to use other microbial solutions (e.g., CeMBio) can bypass these steps that were specifically performed to avoid contaminating new SFNH set-ups from extraneous nematodes.

Our SFNH mesocosm was able to simulate an environment that is similar to the reported habitats of *C. elegans*. In our ES assay, we showed a trend in worm population where it increases from a 6 days-old worm population (12D) to a 15 days-old worm population (21D) (Figure 7C). The Rhabditidae family, where *C. elegans* belongs, is well known for its short generation time with booming population dynamics and high reproduction rate (Bongers and Bongers, 1998; Félix and Duveau, 2012). This drastic increase in population is observed when there is high microbial activity in its habitat. This relationship between bacteria and nematode was also observed in agroecosystems, where Rhabditidae is present when there is a high-nutrient flush, resulting in high bacterial populations in enriched soils (Bongers and Bongers, 1998). This is similar to what we observed in our ES assay, where an increase in worm population was observed as the apple further decomposes. We hypothesize that the proliferation of microbial community takes place as the apple further decays, providing abundant bacterial feed for the nematode and thus the “boom” in the worm population. It is, however, important to note that we also observed an increase in variation as the incubation time increases. Our hypothesis is that this phenomenon illustrates ecology in its truest nature where a slight difference between environmental factors and microenvironment can result in tremendous variations. However, at this point, we do not know the exact reason for the population variations. In the future, we will examine experiments longer than 21 days and also deeper regions of the soil and apple.

Finally, a comparison of the population growth of worms in SFNH for 6 days and plates for 5 days showed no significant differences. However, the developmental stages of these populations were quite different with much fewer L1–L2 young larvae in the SFNH. The reasons for these differences in development are not known. It appears that reproduction and/or development is slightly slower in SFNH, or possibly many of the L1 larvae died in SFNH. Further investigation is required to delineate these possibilities.

**Figure 9:**
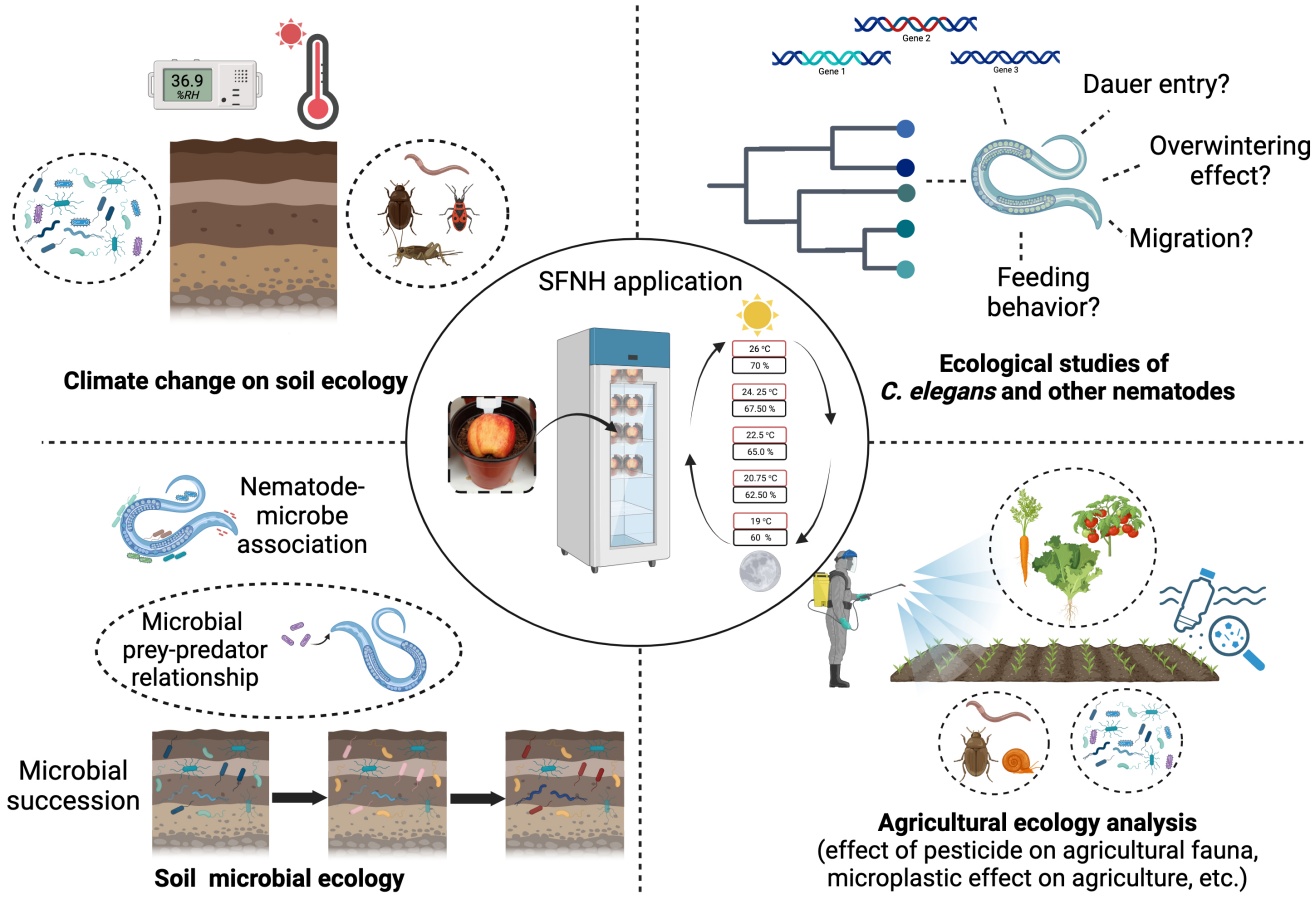
Possible applications of the SFNH protocol. The SFNH protocol application can further the understanding of *C. elegans* biology. This protocol can be used to understand how the nematode behaves and reacts to the factors present in its natural habitat. This protocol can also be used in climatic change effects on soil ecology and the changes in soil microbial ecology. Finally, the agricultural application of this protocol can also help understand the effects of pesticides, microplastic, and other exogenous factors on the natural fauna of the agricultural ecosystem without spreading harm to the environment. Created with BioRender.com.

We have established a novel experimental protocol that allows the cultivation of *C. elegans* in a simulated natural habitat under controlled conditions in the laboratory. Although this protocol cannot and will not replace the cultivation of *C. elegans* in plates for specific experiments, it can help generate more information about *C. elegans* biology outside the Petri dish. It can also be used to further understand *C. elegans* physiology, behavior, and immunity in its natural habitat. In nature, very little is also known about the nematode’s dauer entry and exit behavior; overwintering, migration, and feeding behavior, and this protocol opens an opportunity to further explore the worm’s ecology in a semi-controlled environment. The application of this protocol will also allow laboratory, microbial, and agricultural research that aims to test the effect of pesticides, microplastics, and other exogenous factors on animals because this mesocosm mimics the natural ecology of an animal (Figure 9). The protocol can be adjusted to accommodate molecular experiments such as gene expression, proteomic, or metagenomic analysis, where timing is important. In particular, the timing of recovery of nematodes and/or microbes can be shortened by using a smaller portion of soil, to recover subpopulations of nematodes and microbes. Animal-microbial association in the soil or agricultural environment can also be studied using this protocol and soil microbial succession. These studies can help further the knowledge already available on the dynamics of soil ecology. Effects of long-term temperature changes, humidity, and any extreme climatic conditions on the soil ecosystem can also be studied in the laboratory using this protocol. SFNH mesocosm is a tractable experimental set-up, and we believe that it could be used to expand the existing yet limited understanding that we have about *C. elegans* biology in the wild. We also believe that this protocol can help establish *C. elegans* as a model species for evolutionary and ecological studies in the future.

## Data availability statement

The raw data supporting the conclusions of this article will be made available by the authors, without undue reservation.

## Ethics statement

The manuscript presents research on animals that do not require ethical approval for their study.

## Author contributions

RI: Conceptualization, Formal analysis, Investigation, Methodology, Writing – original draft, Writing – review & editing. JP: Investigation, Methodology, Writing – original draft, Writing – review & editing. J-KH: Investigation, Writing – review & editing. EL: Investigation, Writing – review & editing. TH: Writing – review & editing. JH: Investigation, Resources, Writing – review & editing. TL: Resources, Writing – review & editing. JL: Conceptualization, Formal analysis, Funding acquisition, Investigation, Methodology, Project administration, Resources, Writing – original draft, Writing – review & editing.

## Supplementary material

Supplementary Figure S1 Worm population in deeper areas of the SFNH mesocosm. Worm population found 3 cm beneath the soil surface at Day 12 to Day 21 of the ecological succession. Large populations in this deep layer can occasionally be observed at Day 21. Solid sphere = 1 pot, n=6 pots for each day.
