# Supplementary figures and images for: A simple protocol for cultivating the bacterivorous soil nematode Caenorhabditis elegans in its natural ecology in the laboratory
Source: Front Microbiol. 2024 Feb 27;15:1347797. doi: 10.3389/fmicb.2024.1347797 (PMC10929012; doi:10.3389/fmicb.2024.1347797)

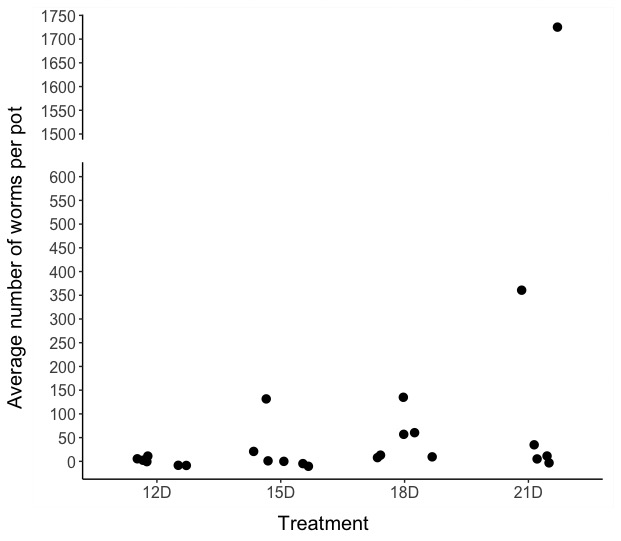

Supplement: Supplementary Figure S1 — Worm population in deeper areas of the SFNH mesocosm. Worm population found 3 cm beneath the soil surface at Day 12 to Day 21 of the ecological succession. Large populations in this deep layer can occasionally be observed at Day 21. Solid sphere = 1 pot, n=6 pots for each day. [file Image_1.JPEG]
